# Comprehensive evaluation of the metabolic effects of porcine CRTC3 overexpression on subcutaneous adipocytes with metabolomic and transcriptomic analyses

**DOI:** 10.1186/s40104-021-00546-6

**Published:** 2021-03-03

**Authors:** Jiaqi Liu, Jie Li, Wentao Chen, Xintao Xie, Xingang Chu, Teresa G. Valencak, Yizhen Wang, Tizhong Shan

**Affiliations:** 1 College of Animal Sciences, Zhejiang University, Hangzhou, China; 2 Key Laboratory of Molecular Animal Nutrition (Zhejiang University), Ministry of Education, Hangzhou, China; 3 Key Laboratory of Animal Feed and Nutrition of Zhejiang Province, 866 Yuhangtang Road, Hangzhou, China

**Keywords:** CRTC3, Energy metabolism, Metabolomics, Overexpression, Subcutaneous adipocytes, Transcriptomics

## Abstract

**Background:**

Meat quality is largely driven by fat deposition, which is regulated by several genes and signaling pathways. The cyclic adenosine monophosphate (cAMP) -regulated transcriptional coactivator 3 (CRTC3) is a coactivator of cAMP response element binding protein (CREB) that mediates the function of protein kinase A (PKA) signaling pathway and is involved in various biological processes including lipid and energy metabolism. However, the effects of CRTC3 on the metabolome and transcriptome of porcine subcutaneous adipocytes have not been studied yet. Here, we tested whether porcine CRTC3 expression would be related to fat deposition in Heigai pigs (a local fatty breed in China) and Duroc×Landrace×Yorkshire (DLY, a lean breed) pigs *in vivo*. The effects of adenovirus-induced CRTC3 overexpression on the metabolomic and transcriptomic profiles of subcutaneous adipocytes were also determined *in vitro* by performing mass spectrometry-based metabolomics combined with RNA sequencing (RNA-seq).

**Results:**

Porcine CRTC3 expression is associated with fat deposition *in vivo.* In addition, CRTC3 overexpression increased lipid accumulation and the expression of mature adipocyte-related genes in cultured porcine subcutaneous adipocytes. According to the metabolomic analysis, CRTC3 overexpression induced significant changes in adipocyte lipid, amino acid and nucleotide metabolites *in vitro*. The RNA-seq analysis suggested that CRTC3 overexpression alters the expression of genes and pathways involved in adipogenesis, fatty acid metabolism and glycerophospholipid metabolism *in vitro*.

**Conclusions:**

We identified significant alterations in the metabolite composition and the expression of genes and pathways involved in lipid metabolism in CRTC3-overexpressing adipocytes. Our results suggest that CRTC3 might play an important regulatory role in lipid metabolism and thus affects lipid accumulation in porcine subcutaneous adipocytes.

**Supplementary Information:**

The online version contains supplementary material available at 10.1186/s40104-021-00546-6.

## Background

Pork, which is widely consumed globally, is an important source of animal protein for humans [1, 2]. Methods to further improve pork quality for human consumption have received increasing attention in recent years. Fat deposition influences growth efficiency, pork production and meat quality [3, 4]. The deposition of subcutaneous and visceral fat directly influences backfat thickness and growth efficiency, while the intramuscular fat (IMF) content directly affects meat quality including the flavor, juiciness and tenderness as well as the fatty acid (FA) composition [2, 3]. Thus, an understanding of fat deposition in pigs is critical for improving meat production and quality. Fat deposition is directly determined by the development of adipose tissue, which mainly consists of adipocytes [5]. In mammals, three types of adipocytes have been identified: white, beige and brown adipocytes. Brown and beige adipocytes have many mitochondria and can use up lipids as an energy source, while white adipocytes specialize in coordinating lipid and energy storage [6]. However, brown adipocytes have not been observed in pigs [7, 8]. White adipocytes comprise the majority of cells in white adipose tissue (WAT). WAT stores are split, in subcutaneous (80% to 90% of body fat), visceral (~ 10% of body fat), intramuscular, intrahepatic, intracardiac and intrapancreatic adipose tissue depots [9]. Subcutaneous white adipose tissue (SAT) is a highly dynamic tissue and considered the largest adipose tissue [10]; furthermore, SAT plays important roles in storing excess energy storage and whole-body metabolism [11]. WAT quickly expands through a combination of adipocyte hypertrophy and hyperplasia driven by both genetic, dietary or environmental factors [10]. Adipocyte hyperplasia is the primary determinant of adipose tissue development and is helpful in maintaining normal adipocyte function in the presence of external stressors [10]. An impairment of adipocytes expansion is strongly associated with adipose tissue dysfunction and metabolic disorder [12]. Adipocytes also produce and release several adipokines and metabolites that alter the expression of genes and affect whole-body metabolism [13]. Based on the important roles of the adipocytes and their metabolites in regulating whole-body energy metabolism, the development of adipocytes and their metabolomic and transcriptomic profiles deserve investigation.

The cyclic adenosine monophosphate (cAMP)-protein kinase A (PKA) signaling pathway plays a critical role in cellular and whole-body energy homeostasis [14–16]. In several types of cells, cAMP-PKA signaling is mediated by the transcription factor cAMP response element binding protein (CREB) and its coactivator CREB-regulated transcription coactivators (CRTCs) to regulate the transcription of downstream target genes [16]. CRTC3, a member of the CRTC protein family [16], is expressed at high levels in white adipocytes and is involved in energy metabolism [17]. The global deletion of CRTC3 enhances energy expenditure and protects mutant mice from obesity [16]. Mice with a CRTC3 knockout in brown adipocytes were significantly more cold-tolerant and showed reduced adiposity, whereas mice with adipocyte-specific overexpression of CRTC3 were cold-sensitive and displayed increased fat deposition [18]. Notably, the nuclear localization of CRTC3 affects uncoupling protein 1 (UCP1) expression and energy metabolism in brown adipocytes [19, 20]. In 3T3-L1 adipocytes, the phosphorylation of CRTC3 regulates glucose transporter 4 (GLUT4) expression and glucose uptake [21]. In addition, overexpression of CRTC3 increases the intramuscular triglycerides (TGs) level in murine skeletal muscle cells [14]. However, no studies have been conducted to date characterize the effects of CRTC3 on adipocyte metabolism. Similarly, the metabolomic and transcriptomic profiles of CRTC3-overexpressing porcine adipocytes have not yet been examined.

In our previous studies, we determined the regulatory role of CRTC3 in brown adipocytes and in intestinal epithelial cells [17, 19, 20, 22]. We also studied the expression pattern of CRTC3 in skeletal muscle in Heigai pigs, which is a profitable pig breed in China that is characterized by high fat deposition, a low lean ratio, good meat color and high IMF contents [2]. In the present study, we aimed to compare the breed-specific difference in CRTC3 expression in SAT and visceral adipose tissue (VAT) between Heigai pigs (higher fat breed) and Duroc×Landrace×Yorkshire (DLY) pigs (a lean crossbred pig breed). Moreover, we applied mass spectrometry-based metabolomics combined with RNA sequencing (RNA-seq) to analyze the effect of CRTC3 overexpression on the metabolomic and transcriptomic profiles of porcine subcutaneous white adipocytes. Our study also reveals the metabolic effects of CRTC3 on adipocytes and suggests that CRTC3 might represent a target gene for regulating fat deposition and meat quality in pigs.

## Materials and methods

### Experimental animals and sample collection

All procedures were approved by the University of Zhejiang Institutional Animal Care and Use Committee. For the *in vivo* study, 4 Heigai pigs (~ 8.0 months old ) and 4 DLY (~ 6.5 months old) pigs having attained the slaughter weight were randomly selected to investigate the expression pattern of CRTC3 and its relationship to fat deposition (e.g. backfat thickness) in pigs. The pigs were previously fed a corn-soy basal diet and raised in a common environment at Shandong Chunteng Food Co. Ltd. (Shandong, China). All pigs were fasted for 12 h before sample collection. Immediately after slaughter, the weights of the carcass, lean muscle mass, skin and fat were measured; and the lean, skin and fat ratios were calculated. The backfat thickness was measured at the midline with a sliding caliper. The SAT and VAT samples were immediately collected from the left half of the body, flash frozen in liquid nitrogen and stored at − 80 °C to determine gene and protein expression.

### Cell culture and adenovirus infection

For the *in vitro* study, porcine subcutaneous adipocytes were isolated from 3-day-old DLY pigs using previously published methods [23]. These cells were cultured in Dulbecco’s Modified Eagle’s Medium (DMEM, high glucose, KeyGEN BioTECH, Jiangsu, China) supplemented with 10% fetal bovine serum (FBS, GIBCO, New Zealand) and 100 U of penicillin and streptomycin at 37 °C in a humidified atmosphere of 5% CO_2_. The adenoviruses encoding green fluorescent protein (GFP), pAdM-FH-GFP (the GFP control, abbreviated as CON) and pAdM-FH-GFP-CRTC3 (the CRTC3 overexpression construct, abbreviated as OE) with high transfection efficiency were purchased from Vigene Company (Vigene, Shandong, China). Before adenovirus infection, cells were seeded in 6-well or 12-well plates and cultured with complete high-glucose DMEM (DMEM/F12 + 10% fetal bovine serum + 100 U of penicillin + 100 U of streptomycin) at 37 °C in a humidified atmosphere of 95% air: 5% CO_2_. After reaching confluence, cells were incubated with high glucose DMEM containing the CON and OE adenoviruses (0.25 μL/mL) for 6 h, after which the medium was replaced with complete medium. After 48 h, the cells were used for immunostaining, gene expression, metabolomics and RNA-seq analyses.

### Immunostaining

Porcine subcutaneous adipocytes (48 h post-infection) were fixed with 4% paraformaldehyde (PFA) and incubated with blocking buffer (2% BSA, 0.2% Triton X-100, 5% goat serum and 0.1% sodium azide in PBS) for 1 h. Then, cells were incubated with a primary antibody against perilipin 1 (Abcam, Cambridge, MA, USA) at 4 °C overnight. After washing with PBS for 3–5 min, the cells were incubated with a secondary antibody and Hoechst for 1 h at room temperature. Images of the immunofluorescence staining were captured as single-channel grayscale images using a ZEISS Axio Observer3 fluorescence microscope with a 20× objective (NA 0.70).

### Protein extraction and western blot assays

Total protein was extracted from SAT, VAT and cultured porcine subcutaneous cells using RIPA lysis buffer (50 mmol/mL Tris-HCl, pH 7.4, 2 mmol/mL EDTA, 1% SDS, 1% Triton X-100, 10% glycerol, 150 mmol/mL NaCl, and 5 g sodium deoxycholate) containing a complete protease inhibitor cocktail. The protein concentrations were determined using a BCA Protein Assay Reagent Kit (Thermo Fisher, Carlsbad, CA, USA). Protein separation and western blot analyses were conducted as previously described [20]. Antibodies against CRTC3 and perilipin 1 were purchased from Abcam (Abcam, Cambridge, MA, USA), and the anti-GAPDH antibody was purchased from Huabio (Zhejiang, China).

### Total RNA extraction and quantitative real-time PCR

Total RNA was extracted from adipose tissues or cultured adipocytes using TRIzol reagent (Thermo Fisher, Carlsbad, CA, USA). The concentration and integrity of the RNA samples were determined using a NanoDrop 2000 instrument (Gene Company Limited, Hong Kong, China). Approximately 2 μg of RNA were subjected to reverse transcription using random primers and the RevertAid First Strand cDNA Synthesis Kit (Thermo Fisher, Carlsbad, CA). qPCR was performed using an Applied Biosystems 7500 Fast Real-Time PCR System (Applied Biosystems, Foster City, CA, USA) and FastStart Universal SYBR Green MasterMix. The PCR cycling parameters were as follows: 40 cycles of 95 °C for 20 s, 60 °C for 20 s, and 72 °C for 20 s. The 2^-∆∆CT^ method was used to analyze the relative changes in gene expression normalized to 18S rRNA expression as an internal control.

### RNA-seq analysis

The RNA-seq analysis was performed by Novogene Biotech (Beijing, China). Briefly, total RNA was isolated from 6 cultured subcutaneous adipocyte samples (3 OE and 3 CON). The RNA purity was determined using a NanoPhotometer® spectrophotometer (IMPLEN, CA, USA). An RNA Nano 6000 Assay Kit and the Bioanalyzer 2100 system (Agilent Technologies, CA, USA) were used to assess RNA integrity. A total amount of 1 μg RNA from each sample was used for RNA sample preparation as the input material. Sequencing libraries were generated using the NEBNext® Ultra™ RNA Library Prep Kit for Illumina® (NEBNext, USA) according to the manufacturer’s recommendations and index sequences were added to attribute sequences to each sample. The clustering of the index-coded samples was performed on a cBot Cluster Generation System using TruSeq PE Cluster Kit v3-cBot-HS (Illumina) according to the manufacturer’s instruction. After cluster generation, the library preparations were sequenced on an Illumina Nova seq platform and 150 bp paired-end reads were generated. Raw sequencing reads were aligned to the porcine genome assembly with CRTC3-overexpressing subcutaneous adipocytes using Hisat2 to generate a database of splice junctions. We selected feature Counts v1.5.0-p3 to count the reads mapped to each gene.

### Metabolomics analysis

Twelve cultured subcutaneous adipocyte samples (6 OE and 6 CON) were used for metabolomics analysis. The metabolomic analysis was performed by Shanghai Biotree Biotech Company (Shanghai, China). All metabolomic data were normalized to internal standards, and the subcutaneous adipocyte samples were also normalized to the mass of each individual sample as a pooled QC sample. LC-MS/MS analyses were performed using a UHPLC system (1290, Agilent Technologies) with a UPLC HSS T3 column coupled to a Q Exactive mass spectrometer (Thermo Fisher). The QE mass spectrometer was used for its ability to acquire MS/MS spectra in information-dependent acquisition (IDA) mode controlled by the acquisition software (Xcalibur 4.0.27, Thermo Fisher), which continuously evaluates the full-scan MS spectrum. Following mass spectrometry and the initial analysis, a metabolite analysis identified the retention time indices and mass spectra were compared with libraries of retention time indices and the mass spectra to identify the extracted mass spectra. The raw data were converted to the mzXML format using ProteoWizard and processed with an in-house program, which was developed using R and based on XCMS. Then, an in-house MS2 database (BiotreeDB) was applied for metabolite annotation. After the principal component analysis (PCA) and orthogonal projections to latent structures discriminant analysis (OPLS-DA) was performed.

### Metabolite classification and pathway enrichment assay

The significantly different metabolites were classified according to their molecular structure signature using an online resource (http://www.hmdb.ca/). LIPID MAPS provided additional details to search for lipid metabolites (http://www.lipidmaps.org/). Pathway enrichment assays, including Gene Ontology (GO) and Kyoto Encyclopedia of Genes and Genomes (KEGG) analyses, were performed to identify which differentially expressed genes (DEGs) were significantly enriched in GO terms or metabolic pathways. GO terms and KEGG pathways with false discovery rates *P* < 0.05 were considered as significantly altered.

### Statistical analyses

Metabolomic data were set at an annotation cutoff of 0.3 and analyzed after log_2_ transformation with a two-tailed Student’s *t*-test (*P-*value) of metabolite ratios. According to the variable influence on projection values obtained from the OPLS-DA model, the significantly altered metabolites were determined based on a threshold (VIP > 1) and the raw *P*-value (*P* < 0.05). RNA-seq data were provided by DESeq2 statistical routines for determining differential expression in digital gene expression data. The *P*-values were adjusted using the Benjamini & Hochberg method. A corrected *P*-value (padj) of 0.05 and absolute fold change of 2 were set as threshold for significantly different expression. For transcriptomic analyses, only hits with false discovery rates (FDR) < 0.05 were considered significantly different. All the correlations were calculated in R using the method “Pearson”. Correlational analyses of metabolomic and transcriptomic data were conducted with the Morpheus tool (https://software.broadinstitute.org/morpheus/) using hierarchical clustering (Pearson correlation). Statistical analyses were performed using the GraphPad Prism 6 software package (Monrovia, CA, USA). Biochemical and metabolomic data were analyzed with SPSS (v.23, SPSS, Inc.). Comparisons were analyzed using unpaired two-tailed Student’s *t*-tests or One-way ANOVAs, as appropriate. Differences among groups were considered statistically significant at *P <* 0.05. Experimental data are presented as means ± SEM.

## Results

### Breed-specific differences in the expression of CRTC3 and fat deposition-related genes

**Fig. 1:**
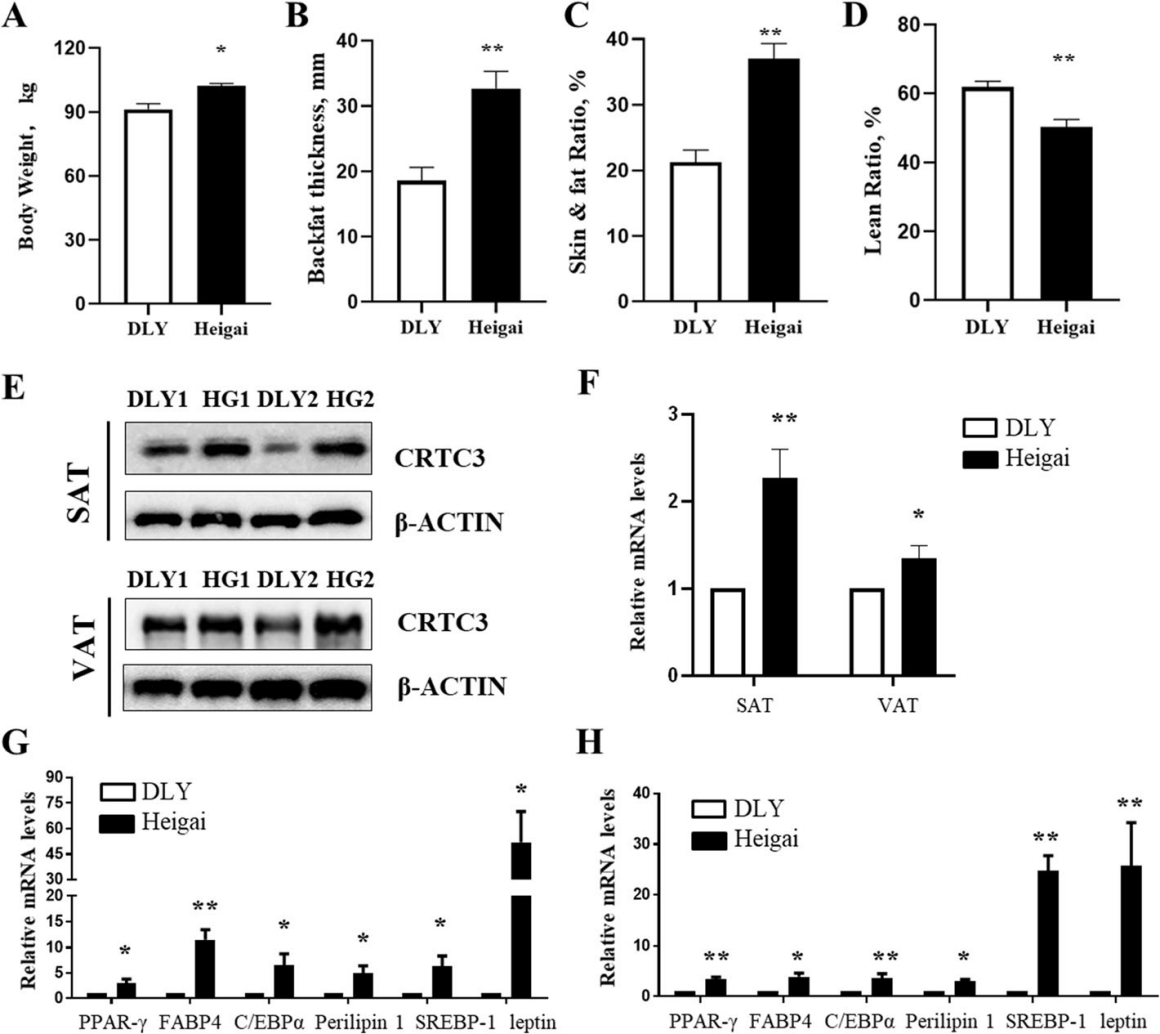
The expression pattern of CRTC3 in SAT and VAT from the lean breed (DLY pigs) and fatty breed (Heigai pigs). **a**, **b**. The body weight (**a**) and backfat thickness (**b**) of DLY and Heigai pigs. **c**, **d**. Comparison of the skin & fat ratio (**c**) and lean ratio (**d**) between DLY and Heigai pigs. **e**, **f**. The protein (**e**) and mRNA (**f**) levels of CRTC3 in SAT and VAT from DLY and Heigai pigs. **g**, **h**. The mRNA levels of adipocyte marker genes in SAT (**g**) and VAT (**h**) from DLY and Heigai pigs. *n* = 4. SEM: standard error of the mean. * *P* < 0.05; **P < 0.01

We first compared the carcass traits of Heigai and DLY pigs (Fig. 1a-d). Our *in vivo* study showed that Heigai pigs had higher body weights (*P* < 0.05) at the same age with a thicker skin (*P* < 0.01) and lower skin & fat ratio (*P* < 0.01) than DLY pigs (Fig. 1a-c). Heigai pigs exhibited a greater adipose deposition capacity and lower lean meat ratio (*P* < 0.01) than DLY pigs (Fig. 1d). Higher levels of the CRTC3 mRNA and protein were detected in the SAT or VAT of Heigai pigs than in the DLY pigs (Fig. 1e-f). Consistent with these findings, the fat deposition marker genes, such as the peroxisome proliferator-activated receptor gamma (*PPARγ*), fatty acid binding protein 4 (*FABP4*), CCAAT/enhancer binding protein alpha (*C/EBPα*), perilipin 1, sterol regulatory element-binding protein 1 (*SREBP-1*), and leptin, were expressed at significantly higher levels in both SAT and VAT of Heigai pigs than in the lean breed (Fig. 1g-h).

### CRTC3 overexpression regulates lipid accumulation in cultured porcine subcutaneous adipocytes

**Fig. 2:**
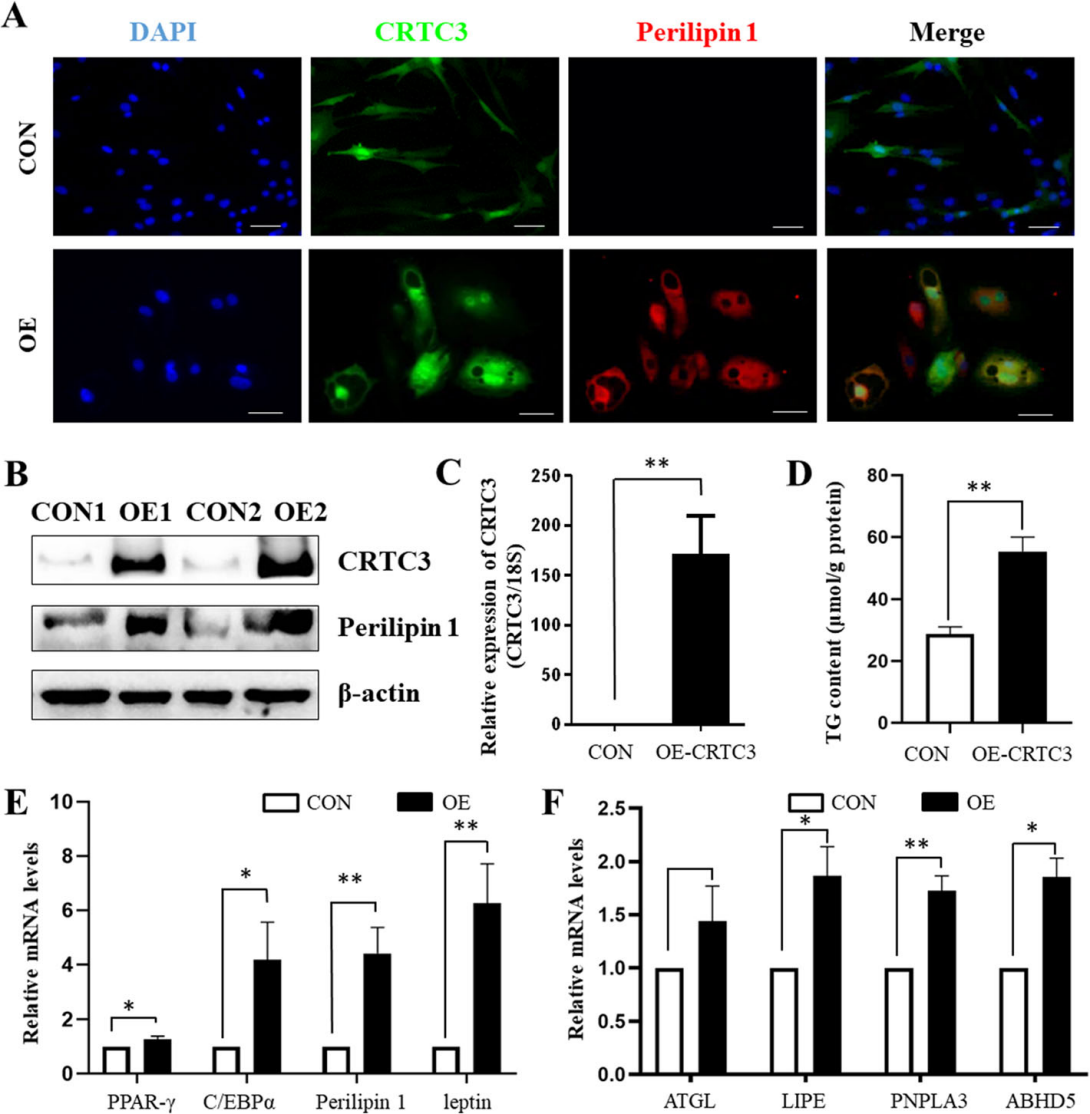
CRTC3 overexpression increases lipid accumulation in white adipocytes. The GFP control (CON) and CRTC3 overexpressing (OE) cells were used for the examination at 48 h after treatment. **a**. Image of immunofluorescence staining for CRTC3 (green) and perilipin 1 (red) in CON or OE adenovirus-treated subcutaneous adipocytes. Nuclei were stained with DAPI (blue). Scale bar, 20 μm. **b**. Protein levels of CRTC3 and perilipin in CON or OE adipocytes. **c**. The relative mRNA levels of CRTC3 in porcine subcutaneous adipocytes. **d**. TG contents in CON or OE adipocytes. **e**. Relative expression of adipose deposition-related gene. **f**. Relative expression of lipolysis-related genes. Relative expression of the target genes in every sample obtained using real-time PCR was normalized to 18S rRNA expression. The data represent the fold change in OE cells relative to the CON cells, which was arbitrarily defined as 1. *n* = 6. SEM: standard error of the mean. * *P* < 0.05; **P < 0.01

To determine the regulatory role of CRTC3 in lipid metabolism *in vitro*, adenovirus-mediated CRTC3 overexpression was induced in porcine subcutaneous adipocytes. Interestingly, CRTC3 overexpression led to noticeable morphological changes in a large number of adipocytes (Fig. 2a). CRTC3 overexpression increased the levels of the perilipin protein, a membrane protein surrounding the lipid droplet (Fig. 2a, b), suggesting that CRTC3 overexpression may increase lipid accumulation in white adipocytes. Indeed, higher expression of the CRTC3 protein and mRNA was detected in the CRTC3 OE group than in the CON group (Fig. 2b, c). In addition, CRTC3 overexpression significantly increased the TG contents in subcutaneous adipocytes (Fig. 2d). Consistent with these findings, the expression of adipogenesis and lipolysis-related genes, including *PPARγ*, *C/EBPα*, perilipin 1, leptin, hormone-sensitive lipase (*HSL*, also known as *LIPE*), was increased in the OE group compared to the CON group (Fig. 2e, f).

### CRTC3 overexpression alters the overall metabolite composition

**Fig. 3:**
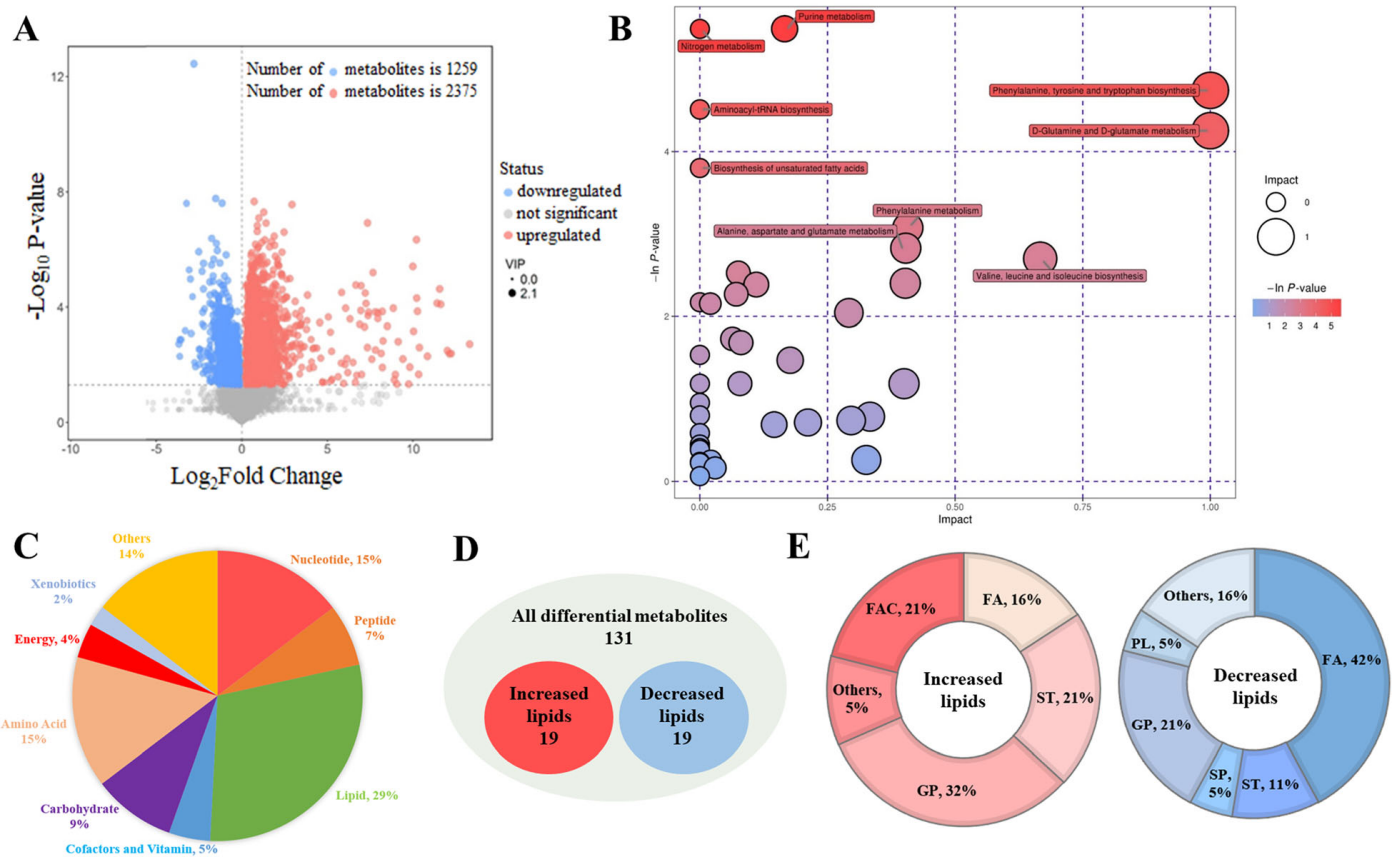
CRTC3 overexpression alters the overall composition of metabolites. The CON and OE adipocytes were harvested at 48 h after treatment, and analyzed using LC-MS/MS**. a**. Volcano plot of pairwise comparisons of all detected ions. The threshold (|log_2_-fold change| > log_2_ 1; *P* value< 0.05) was defined for each significantly changed metabolites. **b**. Significantly altered pathways based on the enrichment and topology analyses. The transverse and vertical dotted lines indicate the value for pathway enrichment and pathway impact, respectivly. Each dot represents a metabolic pathway, and the dot size indicates the significance. **c**. The composition of the significantly altered metabolites. **d**. The altered lipid metabolites in CRTC3-overexpressing adipocytes. **e**. The subclasses of the significantly altered lipid metabolites. FAs, fatty acyls; GP, glycerophospholipids; SP, sphingolipids; ST, steroids; FAC, Fatty acyl carnitines; PL, prenol lipids

To determine the metabolic effects of CRTC3 in adipocytes, we applied an untargeted metabolomic analysis to determine the metabolites in the cultured CON and OE adipocytes. We detected 3634 metabolites, including 2375 upregulated metabolites and 1259 downregulated metabolites (Fig. 3a). The PCA and OPLS-DA plot show a clear separation of the CON and OE groups (Fig. S1A, B). From the OPLS-DA model, 131 significantly altered metabolites were identified by mass spectrum matching in subsequent analyses (Supplementary Table S1). Using functional gene classifications from the KEGG database, we organized the metabolome contents by grouping metabolites into pathways. Metabolites belonging to the same category were placed in adjacent locations to form larger regions and are filled with colors based on the *P*-value displayed in the treemap (Fig. S1C). Treemap analysis was used to confirm the observations from the CRTC3-overexpressing adipocytes, of which glutamine, glutamate, phenylalanine, tyrosine and tryptophan metabolism accounted for the main proportion of the metabolomic changes (Fig. S1C). To compare significance and impact of different pathways individually, we visualized all of the significantly altered metabolite categories using a bubble map, which suggested that the purine and nitrogen metabolism pathways are of equal importance in the metabolomic investigations (Fig. 3b).

To understand the molecular type of specific metabolites, we focused on 131 metabolites sorted by information in the Human Metabolome Database and LIPID MAPS (Fig. 3c). Considerable alterations occurred in the composition and content of metabolites linked to energy and amino acid metabolism in CRTC3-overexpressing cells (Fig. 3c). The significantly altered metabolites included lipid metabolites (29%), amino acid metabolites (15%), nucleotide metabolites (15%), carbohydrate metabolites (9%), peptide metabolites (7%), and cofactors and vitamin metabolites (5%) in OE adipocytes (Fig. 3c). The heatmap displays significantly altered lipid metabolites (Fig. S1D). The significantly altered lipid metabolites included 19 increased lipids and 19 decreased lipids (Fig. 3d). We further classified the lipid metabolites into lipid subclasses (Fig. 3e). In CRTC3 overexpressing adipocytes, the majority of the significantly increased lipid metabolites were glycerophospholipids (GPs, 32%), fatty acyl carnitines (FACs, 21%) and fatty acids (FAs, 16%), and the majority of the significantly decreased were FAs (42%) and GPs (21%) (Fig. 3e). In addition, 11 FA metabolites and 10 GP metabolites were significantly altered in CRTC3-overexpressing cells (Fig. S1E). We also ranked differentially altered GPs according to their fold change and categorized them into lipid subclasses for subsequent integration into the pathway analysis (Fig. S1F). These findings indicate that CRTC3 overexpression induced considerable alterations in the composition and content of metabolites, particularly in the lipid metabolites involved in FA and GP metabolism in porcine subcutaneous adipocytes *in vitro*.

### CRTC3 overexpression alters the expression of genes in metabolic pathways

**Fig. 4:**
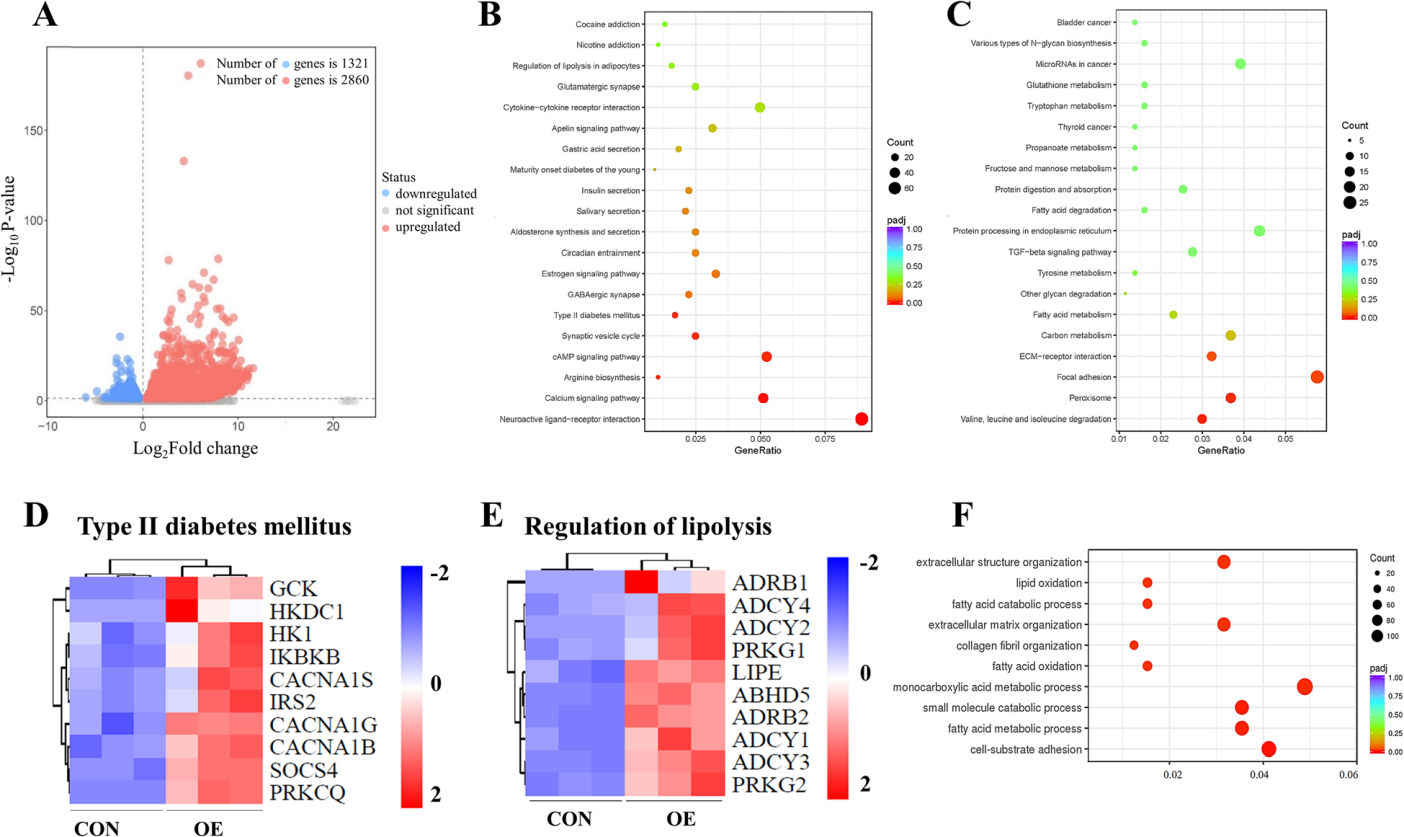
CRTC3 overexpression induces gene programs involved in metabolism. The CON and OE adipocytes were harvested at 48 h after treatment, and RNA was purified for RNA-seq. **a**. Volcano plot of the altered genes in OE versus CON adipocytes. The threshold (|log_2_-fold change| > log_2_ 1.5; padj< 0.05) was defined as a significant change. **b**. The upregulated genes were analyzed by performing a statistical analysis of the KEGG pathway enrichment scatter plot (top 20 genes). The triangle size indicates the significance and the corresponding significance values are displayed as log_10_ (*P-*value). **c**. The downregulated genes were analyzed using the KEGG pathway enrichment statistical scatter plot (top 20 genes). **d, e**. Heatmap of relative expression of selected T2D mellitus- (**d**) and lipolysis-related (**e**) genes from the RNA-seq dataset. **f**. GO enrichment analysis (top 10 genes). CON represents the control group, and OE represents the CRTC3 overexpression group

To explore how the adipocyte metabolites were altered upon CRTC3 overexpression, we applied RNA-seq to map the transcriptional changes and metabolism-related pathways in adipocytes in response to CRTC3 overexpression. The PCA plot showed distinct clusters for the OE and CON groups (Fig. S2A). We found 4181 differentially expressed genes, of which 2860 were upregulated and 1321 were downregulated upon CRTC3 overexpression (Fig. 4a; Supplementary Table S2). As shown in the RNA-seq results, CRTC3 overexpression significantly increased the expression of genes involved in the CRTC3 and cAMP signaling pathway (Fig. 4b; Fig. S2B; Supplementary Table S2). KEGG pathway analysis showed that CRTC3 overexpression also upregulated genes that were involved in calcium signaling, estrogen signaling pathways, type II diabetes mellitus, and cytokine-cytokine receptor interaction signaling pathways (Fig. 4b). In addition, CRTC3 overexpression decreased the expression of genes related to peroxisome, carbon metabolism, FAs metabolism, FAs degradation, tyrosine metabolism, protein digestion and absorption, and glutathione metabolism signaling pathways (Fig. 4c). Correspondingly, functional enrichment analyses of the GO pathways revealed several significantly enriched metabolic pathways in CRTC3-overexpressing cells (Fig. S2C).

Our heatmap results showed that CRTC3 overexpression significantly increased the expression of genes related to the risk of type II diabetes mellitus in CRTC3-overexpressing adipocytes (Fig. 4d). Consistent with our qPCR results, RNA-seq results showed that overexpression of CRTC3 significantly increased the expression of adipogenesis-related genes including the key transcription factor *C/EBPα* (Fig. S2D) and the genes related to and lipolysis in adipocytes, suggesting a regulatory effect of CRTC3 on lipid metabolism in adipocytes (Fig. 4e). Importantly, the pathways in which downregulated genes were enriched correlated with lipid metabolism pathways including FA metabolic, FA catabolic, and lipid oxidation pathways based on the GO pathway analysis (Fig. 4f). In addition, CRTC3 overexpression significantly altered the expression of genes related to amino acid metabolism (Fig. S3A), carbohydrate metabolism (Fig. S3B) and nucleotide metabolism (Fig. S3C). Thus, CRTC3 is involved in regulating metabolic signaling pathways, particularly adipogenesis and lipid metabolism signaling pathways.

### CRTC3 overexpression regulates FA and GP metabolism by modulating their related metabolic pathways

**Fig. 5:**
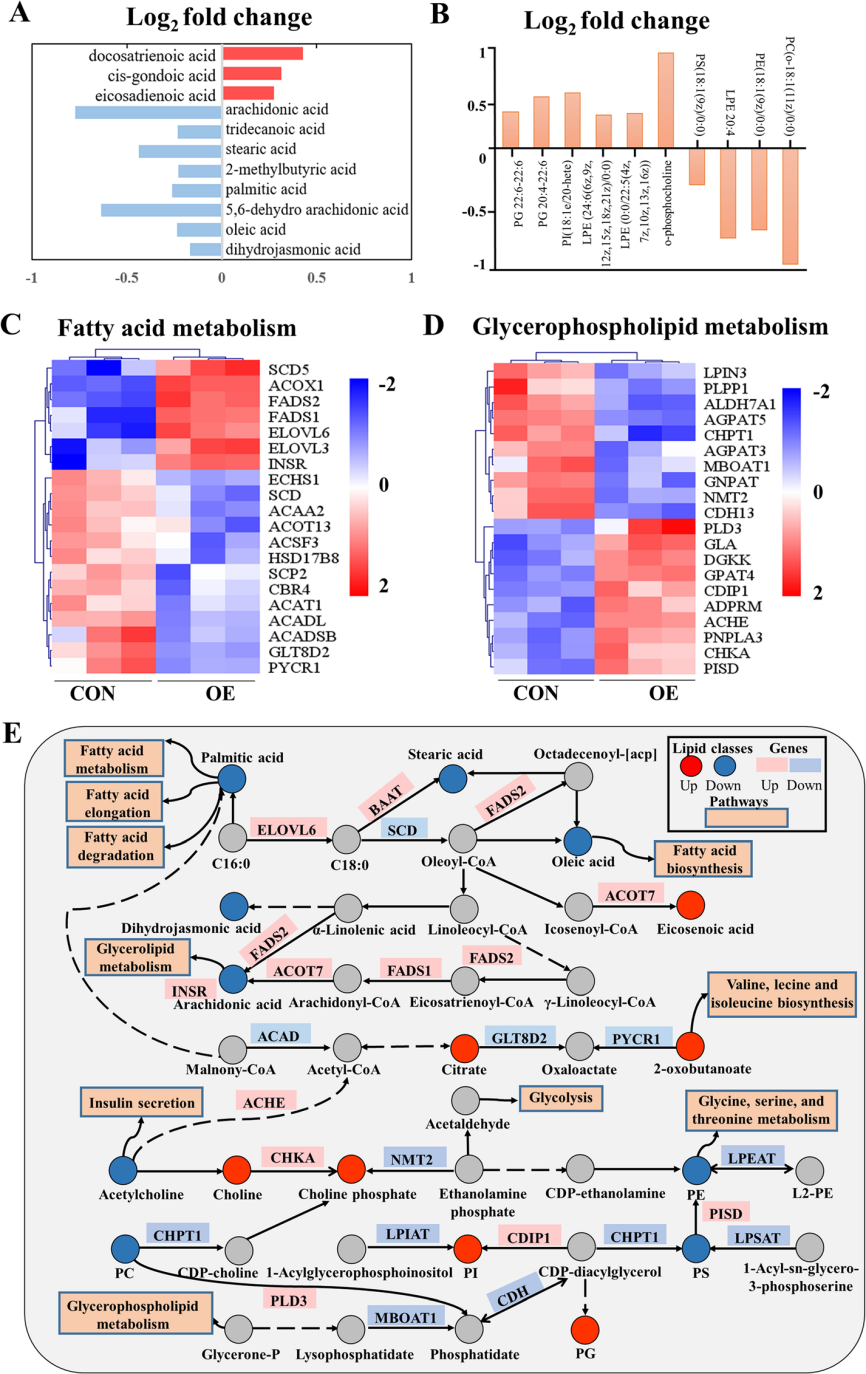
CRTC3 overexpression regulates FA and GP related metabolic pathways. **a**, **b**. The log_2_-fold changes in FA metabolites (**a**) and GP metabolites (**b**) following CRTC3 overexpression. Metabolites are ranked according to *P* value. **c**, **d**. Heatmaps showing the selected differentially expressed genes (DEGs) involved in FA (**c**) and GP (**d**) metabolism in CRTC3-overexpressing adipocytes. **e**. The selected metabolic reactions of lipids (FAs and GPs) from the KEGG analysis, along with interaction of altered lipid metabolites and related genes that were significantly regulated in adipocytes overexpressing CRTC3. Metabolites are indicated by circles (|log_2_-fold change| > log_2_ 1; *P* value< 0.05), and genes are indicated by squares (|log_2_-fold change| > log_2_ 1.5; padj< 0.05). Red indicates an increase or upregulation, blue indicates a decrease or downregulation, and gray indicates undetected. Reaction directions are indicated according to KEGG Mapper (the arrows do not necessarily correspond to reaction reversibility). The dashed lines represented an indirect connection

We further probed subclasses of lipid metabolites that were regulated by CRTC3 overexpression. In adipocytes, overexpression of CRTC3 induced significant increases in the levels of fatty acyls, such as (*R*)-stearoylcarnitine, elaidic carnitine, pivaloylcarnitine and acetylcarnitine (Fig. 5a). Among long-chain unsaturated FAs, the levels of eicosadienoic acid and *cis*-gondoic acid were increased in CRTC3-overexpressing adipocytes (Fig. 5a). Lower levels of arachidonic acid, stearic acid, palmitoleic acid and dihydrojasmonic acid were detected in CRTC3-overexpressing cells (Fig. 5a). Moreover, CRTC3 overexpression regulated GP metabolism (Fig. 5b). The level of *O*-phosphocholine that participates in GP metabolism was significantly increased upon CRTC3 overexpression (Fig. 5b). The levels of FA-containing GPs including PC 18:1 and LPE 20:4 were decreased in the OE group (Fig. 5b).

**Fig. 6:**
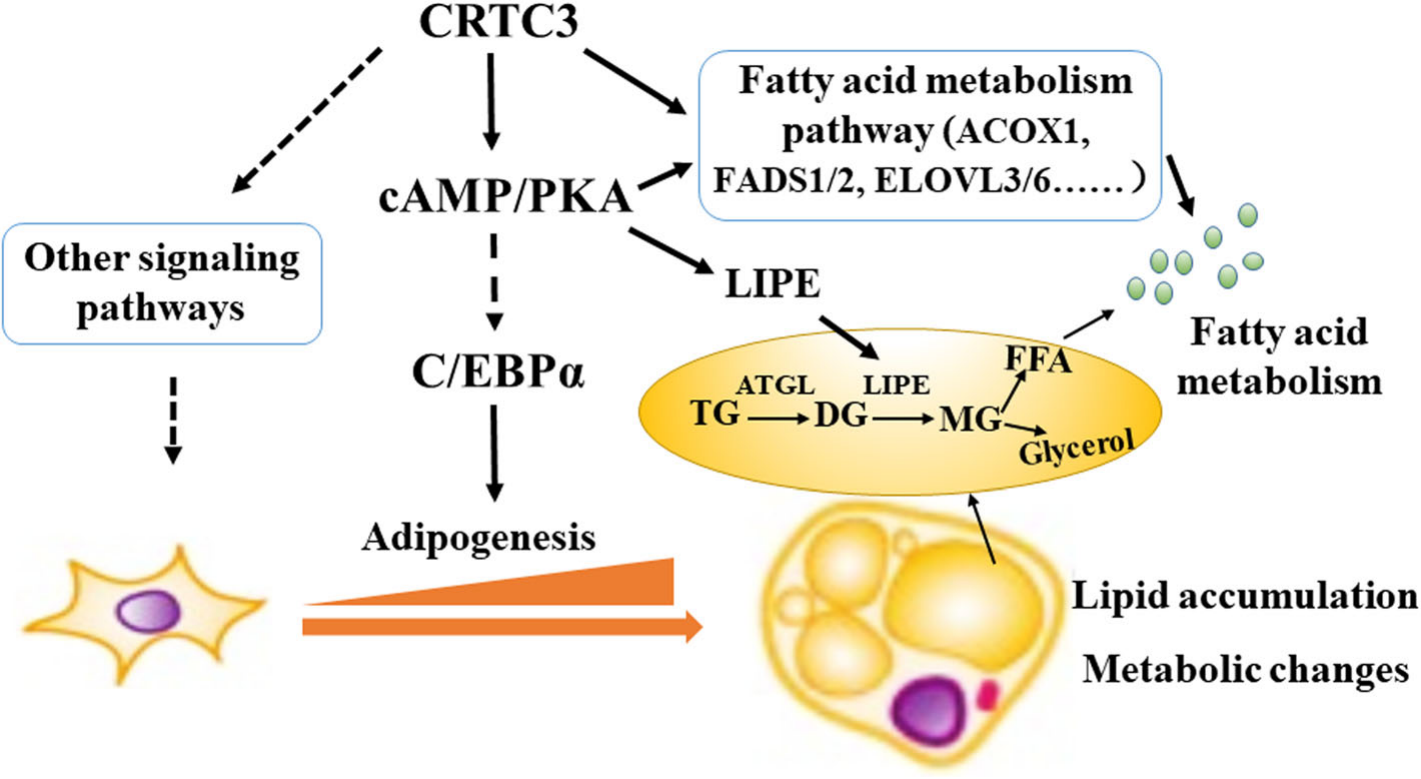
Working model of the metabolic effects of CRTC3 on porcine subcutaneous adipocytes. Using a combination of transcriptomic and metabolomic analyses, overexpression of CRTC3 was shown to activate C/EBPα, the cAMP/PKA and FA metabolism and other signaling pathways to regulate adipogenesis, lipolysis and FA metabolism and subsequently induce lipid accumulation and metabolic changes in porcine adipocytes. Arrows with a dashed line indicate that the regulatory effect must be confirmed, arrows with a solid line indicates direct regulation or a direct connection, and the orange arrow indicates the process of adipogenesis

To identify the signaling pathways involved in FA and GP metabolism, we selected metabolite related genes from the KEGG pathways using a bioinformatics analysis methods (Fig. 5c, d). The heatmap shows that CRTC3 overexpression significantly altered the expression of FA metabolism-related genes including fatty acid desaturase 1 (*FADS1*), stearoyl-CoA desaturase (*SCD*) and acyl-CoA oxidase 1 (*ACOX1*) (Fig. 5c), and the GP metabolism-related genes including patatin like phospholipase domain containing 3 (*PNPLA3*), membrane bound o-acyltransferase domain containing 1 (*MBOAT1*), phospholipase D family member 3 (*PLD3*), and cell death inducing P53 target 1 (*CDIP1*) (Fig. 5d). Moreover, we verified the expression of several lipid metabolism-related genes in samples obtained from our *in vivo* and *in vitro* experiments (Fig. S4). Consistent with the RNA-seq results, higher expression of the FA and GP metabolism-related genes (e.g. *ACOX1*, *FADS2* and *PNPLA3*) was observed in the SAT and VAT from Heigai pigs (Fig. S4A, B). However, the lipolysis-related genes such as adipose triglyceride lipase (*ATGL*, also known as *PNPLA2*) and *HSL* were expressed at lower levels in Heigai pigs than in the lean breed (Fig. S4A, B). Likewise, our qPCR results confirmed that the fatty acid metabolism and lipolysis- related genes (e.g. *ACOX1*, *FADS1* and *FADS2*) were expressed at higher levels in OE adipocytes than in the CON cells (Fig. S4C). Moreover, we conducted a joint analysis of metabolites and the transcriptome and provided an overview of selected lipids and metabolism-related genes from the KEGG analysis (Fig. 5e). The significantly altered genes that play essential roles in different steps of lipid metabolism may explain the changes in lipid metabolites in CRTC3-overexpressing adipocytes (Fig. 5e). Taken together, CRTC3 overexpression drives lipid accumulation and metabolism through regulating the cAMP signaling pathway and the expression of genes related to adipogenesis, lipolysis and fatty acid metabolism, including *C/EBPα*, *HSL* and *ACOX1*, in porcine subcutaneous adipocytes (Fig. 6).

## Discussion

CRTC3 plays important roles in regulating energy metabolism and the expression of genes associated with fat deposition and obesity [16, 24, 25]. Here, the *in vivo* study showed higher expression of CRTC3 in SAT and VAT from the Heigai (fatty) pig breed than in the lean breed, suggesting that CRTC3 expression is indeed associated with fat deposition in pigs. In our *in vitro* study, overexpression of CRTC3 increased lipid accumulation and adipogenesis-related gene expression in cultured subcutaneous white adipocytes. Moreover, we identified the effects of CRTC3 overexpression on adipocyte metabolism and provided metabolomic and transcriptional signatures of white adipocytes overexpressing CRTC3.

We observed higher expression levels of CRTC3 in SAT and VAT from Heigai pigs than DLY pigs. Likewise, CRTC3 expression has been shown to be associated with IMF content in pigs [2]. The genomic characterization of the CRTC3 gene might be associated with meat quality and fat deposition in pigs [26]. Previous studies demonstrated that CRTC3 is expressed at high levels in adipose tissues from different locations [16, 22], and its expression is linked to skeletal muscle fat deposition [2, 14], obesity and energy metabolism [16, 18, 20, 25]. These findings suggest that CRTC3 plays an important regulatory role in fat deposition in mammals. Indeed, our *in vitro* study revealed that adenovirus-mediated CRTC3 overexpression increases lipid accumulation in porcine subcutaneous adipocytes. Consistent with this finding, CRTC3 overexpression also increased lipid accumulation in IMF adipocytes [2] and skeletal muscle [14]. Although it did not affect the white adipocyte number, CRTC3 deletion decreased the adipocyte size in white adipose tissue [16]. Our results, together with the previous findings suggest that CRTC3 expression is related to fat deposition *in vivo* and lipid accumulation *in vitro*.

We concluded that CRTC3 overexpression leads to marked changes in the composition and content of metabolites, including FA, GP, amino acid, carbohydrate, and nucleotide metabolites in cultured porcine adipocytes. Long-chain n-6 polyunsaturated fatty acid (PUFAs) promote adipogenesis and the expression of lipogenic genes and may lead to several metabolism-related health risks [27]. Notably, overexpression of CRTC3 decreased the levels of n-6 PUFAs (arachidonic acid) but increased the levels of n-3 PUFAs (docosatrienoic acid and eicosadienoic acid). Supplementation with n-3 PUFAs exerts more beneficial effects on protecting against obesity by decreasing plasma TG levels [28]. In animal production, dietary PUFAs supplementation or an adjustment of n-6/n-3 PUFA ratio has been reported to regulate meat quality and fatty acid deposition in pigs [29–31]. We also detected high levels of choline in CRTC3-overexpressing cells, which is required for the conversion to acetylcholine and the formation of phospholipid membranes [32]. In addition, oleic acid, a monounsaturated fatty acid (MUFA), is coordinated with saturated FAs including palmitic acid and stearic acid, to modulate FA biosynthesis and oxidation. Among these lipids, decreases in palmitic acid and oleic acid levels attenuate the adipokine-related insulin signaling pathway including a greater release of non-esterified FAs and likely promoting cellular dysfunction associated with ectopic lipid deposition [33]. CRTC3 overexpression also increased the levels of fatty acyls, including (R)-stearoylcarnitine, elaidic carnitine, pivaloylcarnitine and acetylcarnitine, which facilitate the translocation of long-chain FAs from the cytosol into the mitochondrial matrix for subsequent β-oxidation [34]. Moreover, CRTC3 overexpression regulated GP metabolism, which is essential for the formation of cellular membranes, including the cellular morphological membrane fluidity, dynamics, and homeostasis [35]. In addition, CRTC3 overexpression altered the levels of tricarboxylic acid (TCA) cycle intermediates, including fumarate, citrate and malate, and the concentrations of these intermediates are positively associated with obesity [36]. These results suggest that CRTC3 plays an essential role in adipocyte metabolism particularly in lipid and energy metabolism.

We explored the signaling pathways that were involved in mediating the metabolic effect of CRTC3 overexpression on porcine subcutaneous adipocytes using RNA-seq. The significantly altered genes and pathways are related to the metabolism of FAs, amino acids, carbohydrates and nucleotides. Notably, overexpression of CRTC3 influenced gene programs involved in cAMP, adipogenesis, lipolysis, and FA and GP metabolism. *C/EBPα* and *PPARγ* are key transcription factors that control adipogenesis [37, 38]. According to our qPCR and RNA-seq results, CRTC3 overexpression significantly increased the expression of *C/EBPα*, suggesting that CRTC3 regulates adipogenesis by controlling *C/EBPα* expression in porcine adipocytes. CRTC3 overexpression upregulates the genes involved in the cAMP-PKA signaling pathway, which is crucial for energy and lipid metabolism [39]. The cAMP-PKA pathway modulates the expression of downstream target genes, such as *HSL*, a rate-limiting enzyme for diacylglycerol [40] hydrolysis of lipids [41]. Although *HSL* was expressed at high levels in CRTC3-overexpressing adipocytes, the expression of *ATGL*, the rate-limiting enzyme in TG synthesis [42], was not changed in CRTC3-overexpressing cells. Moreover, *HSL* also represents a marker of late adipocyte differentiation and its expression is induced by lipid accumulation and is increased during adipogenesis [38]. In contrast to our findings in CRTC3-overexpressing adipocytes, lower expression levels of *ATGL* and *HSL* were detected in Heigai (fatty) pigs than in the lean breed of pigs. Although the expression pattern was similar, the expression levels of CRTC3 and other adipogenic genes differ *in vivo* and *in vitro*. These differences may occur due to the higher expression of CRTC3 in the CRTC3*-*overexpressing adipocytes *in vitro* compared to adipose tissues *in vivo*. Further studies are needed to determine whether CRTC3 induces higher expression of adipogenic genes *in vivo*. In addition, CRTC3 regulates the expression of *ADCY* family that affects the synthesis of cAMP from ATP and links the regulation of adipose tissue development to adiposity [43, 44]. In addition, CRTC3 overexpression also increased the expression of FA metabolism-related genes including the *ACOX1*, *FADS1/2* and *ELOVL3/6*. Together, these results suggest that CRTC3 overexpression regulates lipid accumulation and metabolism in porcine adipocytes possibly through the adipogenesis- and cAMP-related signaling pathways (Fig. 6). However, the precise regulatory mechanism underlying the metabolic effects of CRTC3 on adipocytes requires further study.

## Conclusion

In conclusion, we studied the breed-specific differences in CRTC3 expression in adipose tissues between fatty and lean breeds of pigs and revealed the metabolic effects of CRTC3 overexpression on adipocytes using the integrative approach of transcriptomics and metabolomics. CRTC3 overexpression accelerates lipid accumulation in adipocytes possibly by regulating adipogenesis, cAMP and lipolysis related signaling pathways (Fig. 6). For the first time, our study provides a comprehensive resource describing the transcriptomic and metabolomic effects of CRTC3 overexpression on porcine subcutaneous adipocytes. Our findings provide new insights that improve our understanding of the molecular signatures regulated by CRTC3 that are involved in lipid and energy metabolism; these insights may become useful for developing strategies to regulate fat deposition and meat quality in pigs.

## Supplementary Information

**Additional file 1: Supplementary Fig. 1**. Identification and classification of significantly altered metabolites in CON and OE adipocytes. **A.** Unsupervised PCA score plot. Purple and blue symbols represent the OE and CON groups, respectively. **B**. Corresponding validation plots of OPLS-DA from the metabolite database. **C**. Treemap of significantly altered pathways. The treemap is shown in as a standard square layout whose area corresponds to the portion of dataset. **D**. Heatmap analysis showing the significantly altered lipid metabolites. **E**. The number of subclasses of lipid metabolites that were significantly changed in the CRTC3 overexpression groups. **F**. Categories of lipid subclasses for selected lipid metabolites.

**Additional file 2: Supplementary Fig. 2**. Multivariate data analysis and quantitative transcriptomic analysis of the expression of selected genes. **A**. Unsupervised PCA score plot. Red and orange symbols correspond to the OE and CON groups, respectively. **B**. Heatmap showing the selected DEGs involved in the cAMP signaling pathway in CRTC3-overexpressing adipocytes. **C**. GO terms of enriched in the total clustered genes. GO terms enriched pathways are categorized as biological processes (BPs), cell components (CCs) and molecular functions (MFs). **D**. Heatmap showing the selected DEGs.

**Additional file 3: Supplementary Fig. 3**. CRTC3 overexpression affects amino acid, carbohydrate and nucleotide metabolic pathways. **A-C**. Heatmaps showing the relative expression of selected genes related to amino acid (**A**), carbohydrate (**B**), and nucleotide (**C**) metabolism from the RNA-seq dataset.

**Additional file 4: Supplementary Fig. 4**. qPCR verification of the significantly altered genes related to lipolysis and fatty acid metabolism identified in the RNA-seq results. **A, B**. The mRNA levels of adipocyte lipolysis related genes in SAT (**A**) and VAT (**B**) from DLY and Heigai pigs. *n* = 4. **C.** The mRNA levels of fatty acid metabolism-related genes in CON or OE adenovirus-treated subcutaneous adipocytes (*n* = 6). SEM: standard error of the mean. * *P* < 0.05; **P < 0.01.

**Additional file 5: Supplementary Table S1.** The significantly altered metabolites upon CRTC3 overexpression.

**Additional file 6: Supplementary Table S2.** The differentially expressed genes upon CRTC3 overexpression.

## Data Availability

Data are available from the authors upon reasonable request.

## Abbreviations

ACOX1: Acyl-CoA oxidase 1
ADCY: Adenylate cyclase
ABHD5: Abhydrolase domain containing 5
ATGL: Adipose triglyceride lipase
ATP: Adenosine triphosphate
cAMP: Cyclic adenosine monophosphate
CREB: Camp response element binding protein
CRTC3: Camp-regulated transcriptional coactivator 3
C/EBPα: CCAAT/enhancer binding protein alpha
FA: Fatty acid
FABP4: Fatty acid binding protein 4
FACs: Fatty acyl carnitines
FADS1: Fatty acid desaturase 1
FDR: False discovery rate
GO: Gene Ontology
GP: Glycerophospholipid
HSL (LIPE): Hormone-sensitive lipase
KEGG: Kyoto Encyclopedia of Genes and Genomes
MBOAT1: Membrane bound o-acyltransferase domain containing 1
MUFA: Monounsaturated fatty acid
OPLS-DA: Orthogonal projections to latent structures discriminant analysis
padj: Corrected *P*-value
PCA: Principal component analysis
PKA: Protein kinase A
PL: Prenol lipids
PLD3: Phospholipase D family member 3
PNPLA3: Patatin like phospholipase domain containing 3
PPARγ: Peroxisome proliferator-activated receptor gamma
PUFA: Polyunsaturated fatty acid
RNA-seq: RNA sequencing
SAT: Subcutaneous adipose tissue
SCD: Stearoyl-CoA desaturase
SP: Sphingolipids
SREBP-1: Sterol regulatory element-binding protein 1
TCA: Tricarboxylic acid
UCP1: Uncoupling protein 1
VAT: Visceral adipose tissue
WAT: White adipose tissue
