# Supplementary figures and images for: Comprehensive evaluation of the metabolic effects of porcine CRTC3 overexpression on subcutaneous adipocytes with metabolomic and transcriptomic analyses
Source: J Anim Sci Biotechnol. 2021 Mar 3;12:19. doi: 10.1186/s40104-021-00546-6 (PMC7927250; doi:10.1186/s40104-021-00546-6)

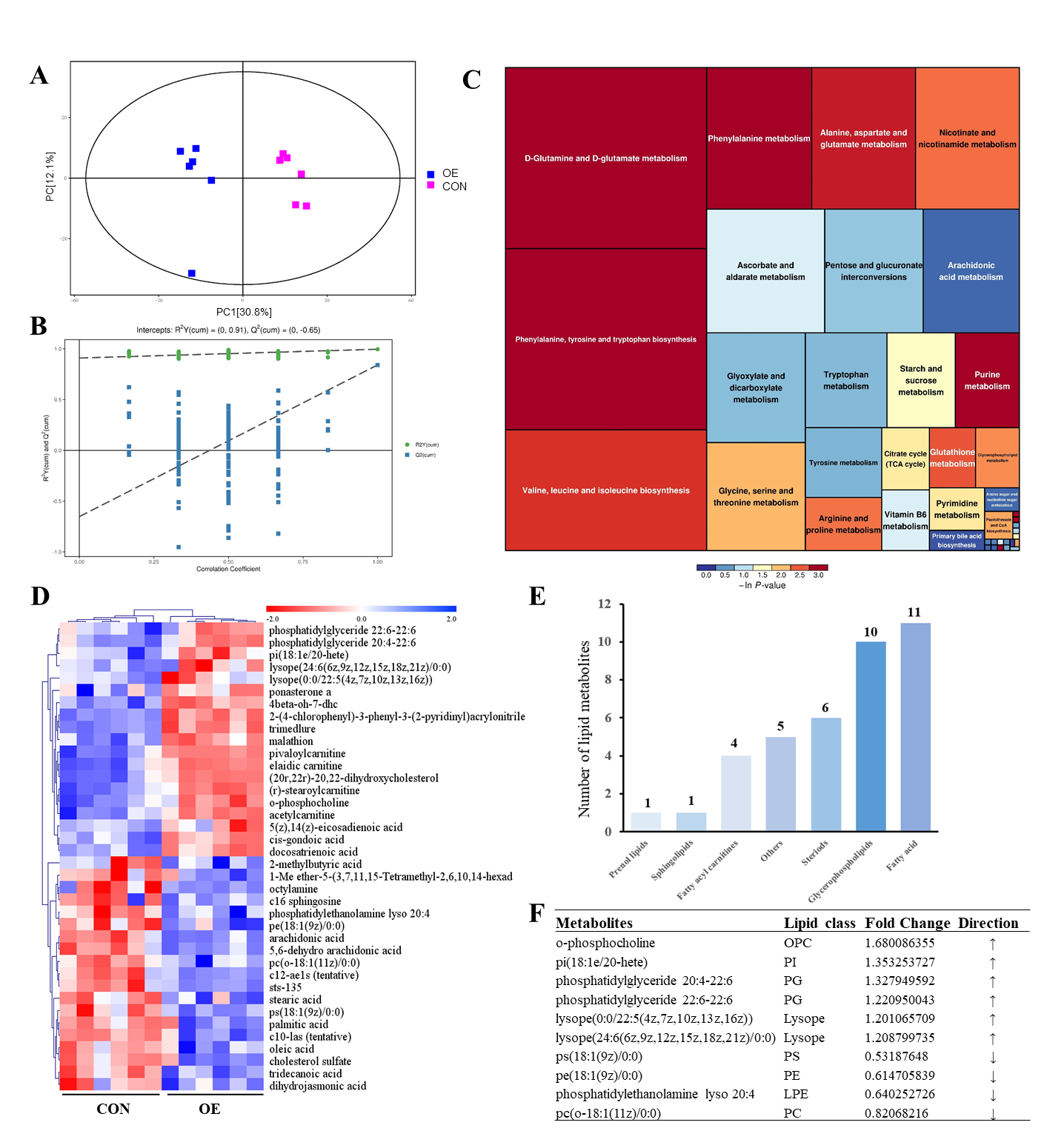

Supplement: Supplementary file 1 — Additional file 1: Supplementary Fig. 1. Identification and classification of significantly altered metabolites in CON and OE adipocytes. A. Unsupervised PCA score plot. Purple and blue symbols represent the OE and CON groups, respectively. B. Corresponding validation plots of OPLS-DA from the metabolite database. C. Treemap of significantly altered pathways. The treemap is shown in as a standard square layout whose area corresponds to the portion of dataset. D. Heatmap analysis showing the significantly altered lipid metabolites. E. The number of subclasses of lipid metabolites that were significantly changed in the CRTC3 overexpression groups. F. Categories of lipid subclasses for selected lipid metabolites. [file 40104_2021_546_MOESM1_ESM.tif]

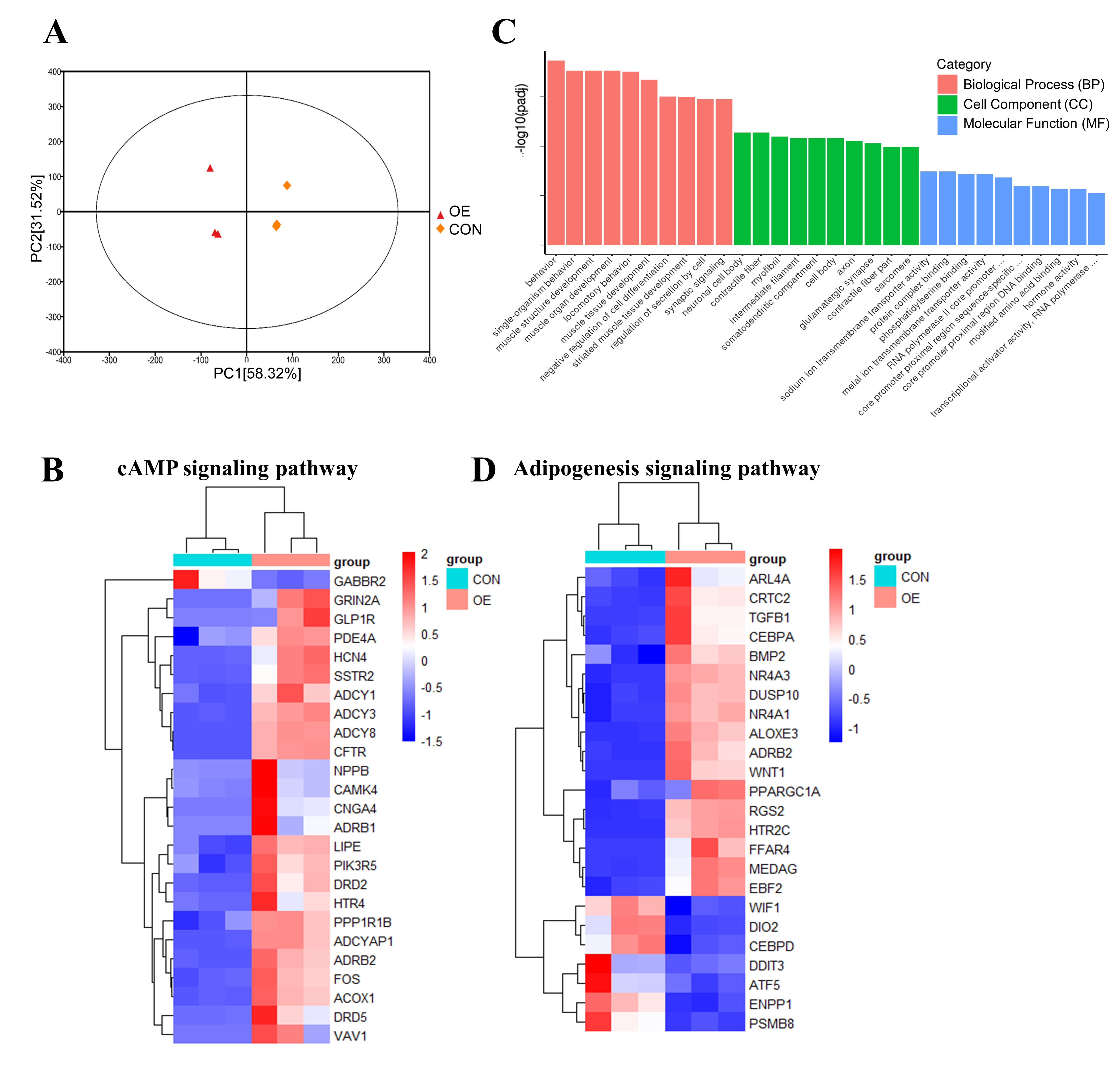

Supplement: Supplementary file 2 — Additional file 2: Supplementary Fig. 2. Multivariate data analysis and quantitative transcriptomic analysis of the expression of selected genes. A. Unsupervised PCA score plot. Red and orange symbols correspond to the OE and CON groups, respectively. B. Heatmap showing the selected DEGs involved in the cAMP signaling pathway in CRTC3-overexpressing adipocytes. C. GO terms of enriched in the total clustered genes. GO terms enriched pathways are categorized as biological processes (BPs), cell components (CCs) and molecular functions (MFs). D. Heatmap showing the selected DEGs. [file 40104_2021_546_MOESM2_ESM.tif]

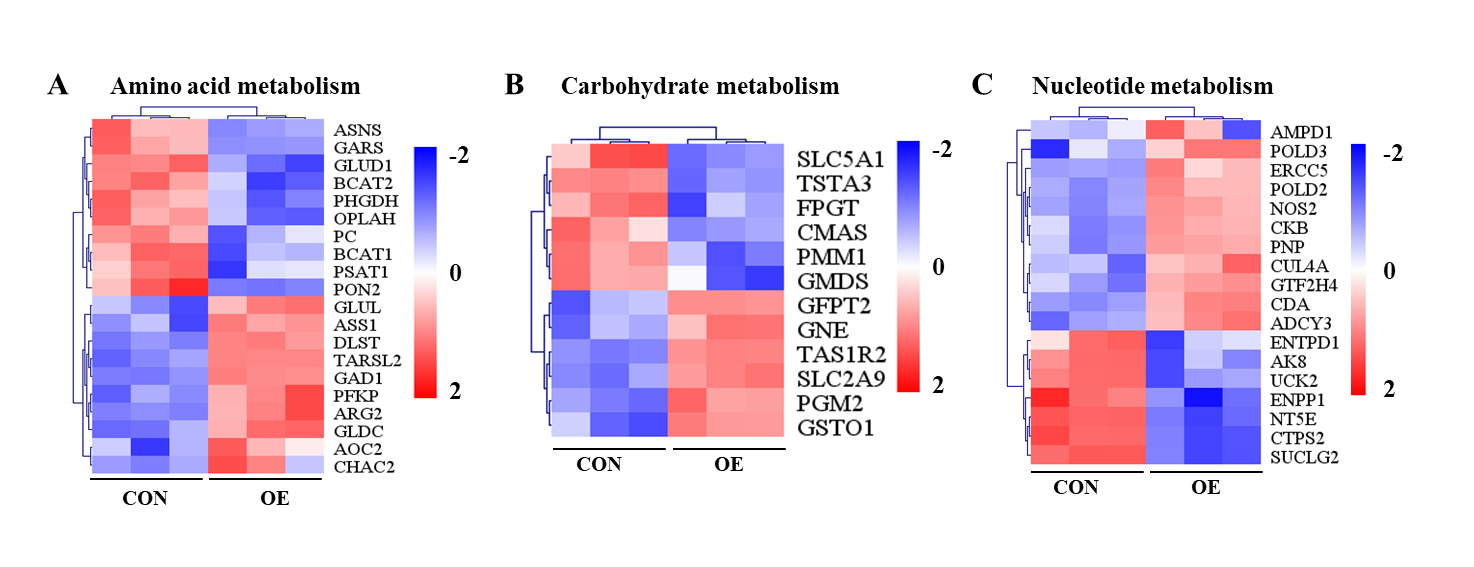

Supplement: Supplementary file 3 — Additional file 3: Supplementary Fig. 3. CRTC3 overexpression affects amino acid, carbohydrate and nucleotide metabolic pathways. A-C. Heatmaps showing the relative expression of selected genes related to amino acid (A), carbohydrate (B), and nucleotide (C) metabolism from the RNA-seq dataset. [file 40104_2021_546_MOESM3_ESM.tif]

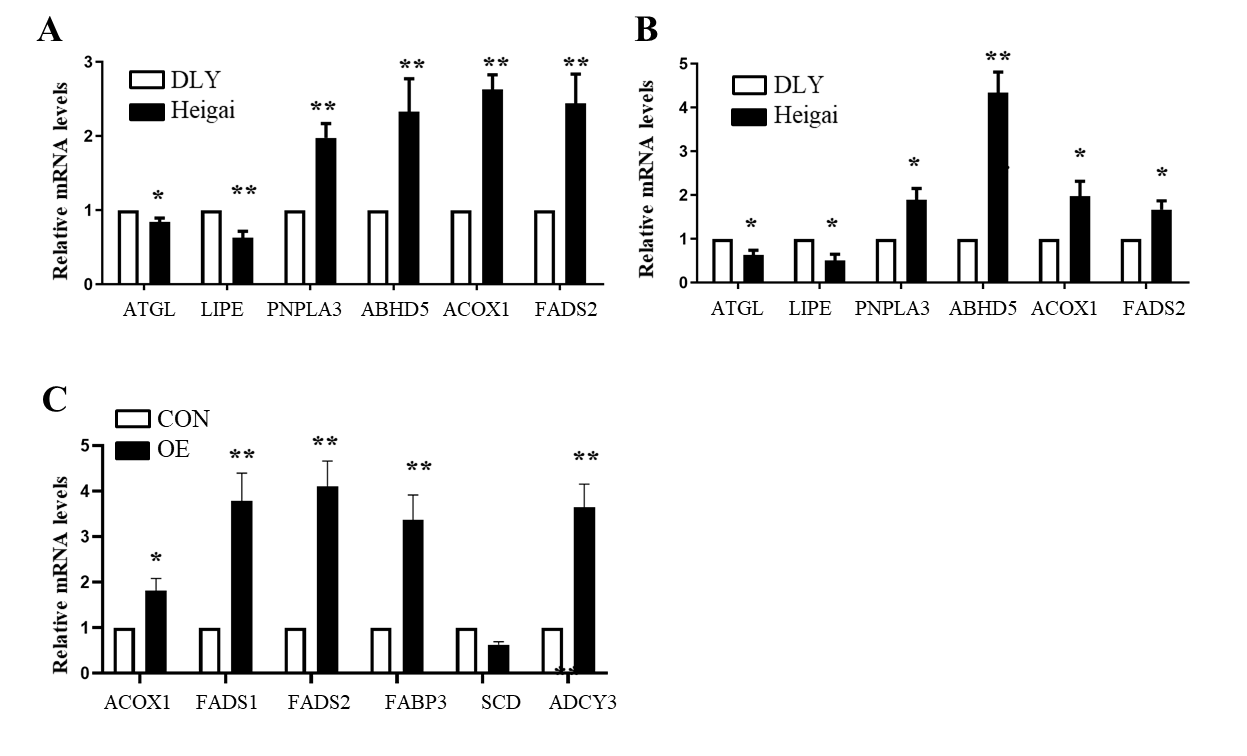

Supplement: Supplementary file 4 — Additional file 4: Supplementary Fig. 4. qPCR verification of the significantly altered genes related to lipolysis and fatty acid metabolism identified in the RNA-seq results. A, B. The mRNA levels of adipocyte lipolysis related genes in SAT (A) and VAT (B) from DLY and Heigai pigs. n = 4. C. The mRNA levels of fatty acid metabolism-related genes in CON or OE adenovirus-treated subcutaneous adipocytes (n = 6). SEM: standard error of the mean. * P < 0.05; **P < 0.01. [file 40104_2021_546_MOESM4_ESM.tif]
